# An Evolutionary Metric for Estimating PhyloAges from Bulk Sequencing of Hematopoietic Stem Cells Reveals the Tempo of Blood Aging in Cancer and Longevity

**DOI:** 10.1007/s00239-025-10296-y

**Published:** 2025-12-26

**Authors:** Jack M. Craig, Ryan M. Tobin, Walter Wolfsberger, Taras K. Oleksyk, Sayaka Miura, Glenn S. Gerhard, Sudhir Kumar

**Affiliations:** 1 Institute for Genomics and Evolutionary Medicine, Temple University, Philadelphia, PA USA; 2 Department of Biology, Temple University, Philadelphia, PA USA; 3 Department of Biological Sciences, Oakland University, Rochester, MI USA; 4 Department of Biology, University of Mississippi, Oxford, MS 38677 USA; 5 Department of Medical Genetics and Molecular Biochemistry, Lewis Katz School of Medicine, Temple University, Philadelphia, PA USA

**Keywords:** Physiological aging, Hematopoietic stem cells, Somatic mutations, Risk prediction

## Abstract

The phylogeny of a person’s hematopoietic stem cells (HSCs) can be used to quantify physiological aging of blood using a phyloAge model based on diversity decay metrics. However, this procedure currently requires accurate HSC genome sequences, which are expensive and time-consuming to obtain. We show that metrics of diversity decay can be derived from the somatic variant frequency spectrum (VFS) using more affordable, routine bulk sequencing, because HSCs evolve without recombination at a clock-like rate. We found that VFS-based models produce phyloAge estimates similar to those derived from HSC genome phylogenies. Customized for protein-coding variation and sequencing read depth, VFS-based HSC phyloAge estimates were, on average, 168 years more than chronological ages in 157 patients with acute myeloid leukemia, consistent with excess HSC aging observed in cancer patients using single cell genome phylogenies. We also tested the hypothesis that variants in cancer driver genes may confer longevity, as they occur in a significant fraction of long-lived individuals. Indeed, HSC phyloAge estimates were significantly lower, consistent with reduced hematologic cancer risk among extremely old individuals. Thus, the new metrics and models broaden the utility of the phyloAge approach, making it feasible and efficient for clinical and research applications.

**Supplementary Information:**

The online version contains supplementary material available at 10.1007/s00239-025-10296-y.

## Introduction

**Fig. 1:**
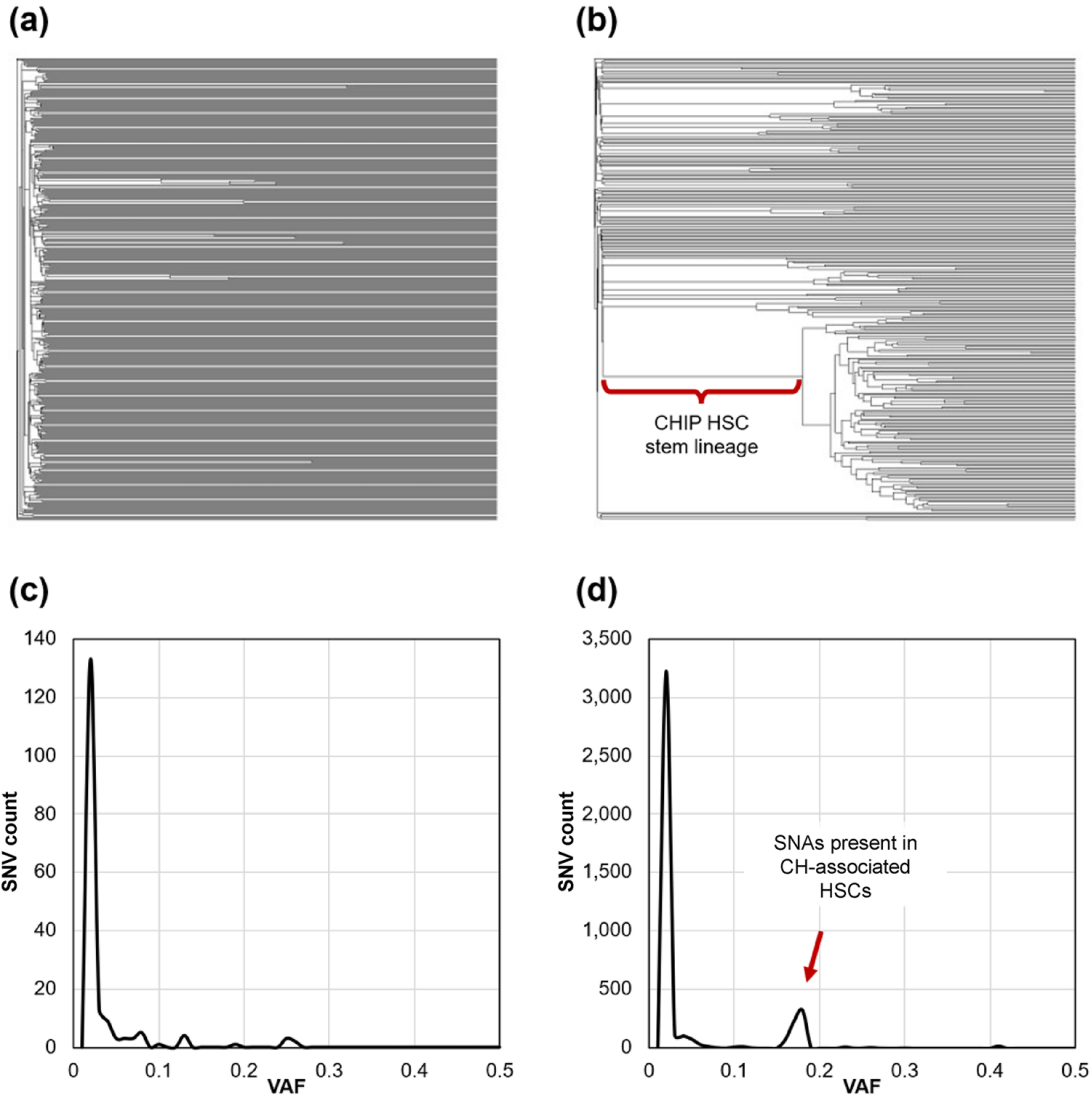
Example phylogenies of HSCs from healthy individuals. **a** A 38-year-old individual with very few bifurcations of HSC lineages following the initial set of diversifications that occurred during embryogenesis. **b** HSC phylogeny of an 81-year-old individual that contained many secondary bifurcations occurred after embryogenesis, resulting in clonal hematopoiesis (CH). The lineage highlighted by the red bracket leads to a large CH event. **c** The VFS in the population of HSCs for the 38-year-old individual in panel **a**. **d** The VFS of the 81-year-old individual from panel **b**, where the peak indicated by the red arrow consisted of SNAs shared by the HSC clade emanating from the branch indicated in red in panel **b**. Like a folded site frequency spectrum plot, **c** and **d** show the frequency of SNAs carrying a range of VAFs, indicating spikes in high-frequency variants with age

There is a direct relationship between aging and the loss of phylogenetic diversity among the hematopoietic stem and progenitor cells (HSCs/HSPCs) that give rise to differentiated blood cells (Lee et al. 2019; Mejia-Ramirez and Florian 2020; Craig et al. 2024). An adult individual’s HSC lineages originate in fetal development and evolve through the earliest phases of life. At younger ages, distinct HSC lineages are observed as long-tip lineages in an HSC phylogeny (Fig. 1a). Each primary HSC replenishes via asymmetrical cell division and accumulates mutations with time without producing additional offspring lineages. At older ages, some primary HSC lineages bifurcate to establish new clades of secondary HSCs, a process called clonal hematopoiesis (CH; Fig. 1b).

**Fig. 2:**
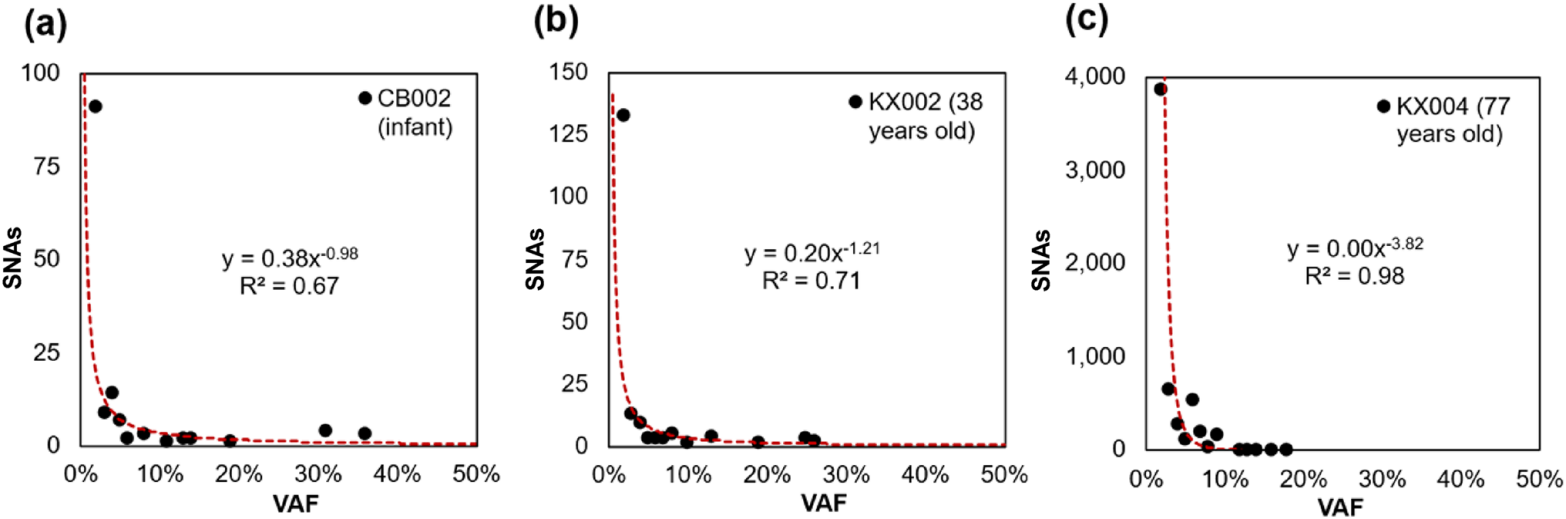
Number of variants with appreciable frequencies (≥ 1%) in **a** an infant, **b** a 38-year-old, and **c** a 77-year-old individual. A steep exponential decay fits this pattern, as shown by red curves and equations in each panel

The presence of CH reduces overall HSC phylogenetic diversity because the genomes of secondary HSCs, which diverged relatively recently, are more similar to one another than those of primary HSCs, which diverged early in development (Lee et al. 2019; Mitchell et al. 2022; Craig et al. 2024). This decline in phylogenetic diversity is consistent with an increased risk of some blood cancers associated with CH (Jaiswal et al. 2014; Jaiswal 2020; Craig et al. 2024). The relationship between phylogeny diversity and age formed the basis for a predictive model of physiological aging gleaned from personal HSC phylogenies, referred to as phyloAge (Craig et al. 2024). Application of the phyloAge model for individuals with myeloproliferative neoplasms (MPN) suggested accelerated HSC aging (Craig et al. 2024). Therefore, phylogenetic methods can detect and quantify the progression of blood cancers independently of driver mutation analysis and standard cytological metrics (Craig et al. 2024). Notably, the phyloAge approach does not require HSC population sizes or use mutation rates. However, reconstructing HSC phylogenies does require accurate genome sequences, as somatic variants accumulate slowly at a rate of ~ 17 single nucleotide alterations (SNAs) per genome per year (Lee-Six et al. 2018; Mitchell et al. 2022). Given the pattern of non-branching evolution in HSCs (Fig. 1a), a vast majority of these SNAs occur in terminal lineages. Indeed, more than 99% of variants have population frequencies < 1% (Fig. 2). This means that when modeling blood health, variants with frequency < 1% should be excluded for quantifying decay in HSC genomic diversity.

With age, some HSCs undergo expansion due to CH, and variants present in those lineages will increase in frequency, as will some new variants acquired during expansion. Thus, the incidence of many higher-frequency variants serves as a biomarker of the decay in phylogeny diversity with age. Indeed, many more high-frequency variants occur in individuals with extensive CH associated with blood cancer (Jaiswal et al. 2014; Toth et al. 2019; Jaiswal 2020; Williams et al. 2022).

Because somatic variants are relatively rare and low-frequency in healthy individuals, HSC genomes need to be accurately sequenced, but routine single-cell sequencing can suffer from significant data sparsity and extensive error (Goswami et al. 2021). Mitchell et al. (2022) and Williams et al. (2022) used an alternative approach in which individual HSCs were first cultured into colonies. Then, DNA from each colony was sequenced. This approach, which we call colony sequencing (colony-seq) to distinguish it from direct single-cell sequencing, achieved high coverage and low base-level error (see Materials and Methods). While effective, colony-seq is time- and resource-intensive, limiting the broader adoption of the phyloAge approach in routine research and clinical investigations.

In contrast, bulk sequencing (bulk-seq) is more affordable and commonly used in studies of blood cancer to identify coding variants and their somatic frequencies in patients. This prompted us to explore advancing the phyloAge approach by developing new metrics to quantify phylogeny decay from a variant frequency spectrum (VFS), which is known to change in response to CH (Watson et al. 2020; Körber et al. 2025). Based on theoretical considerations, we developed novel measures of HSC phylogeny diversity decay computed from VFS, inspired by the insight that somatic cellular evolution occurs without recombination through mitotic cell division. Also, SNA accumulation is known to be clock-like in healthy people as well as blood cancer patients (Lee-Six et al. 2018; Lee-Six and Kent 2020; Craig et al. 2024). Because the new VFS-derived diversity decay metrics showed a strong relationship with their previously developed HSC phylogeny-based counterparts (see Results), we used them to build novel VFS-based models to estimate phyloAge, which we denote phyloAge* to distinguish them from estimates obtained using the HSC genome phylogenies.

Here, we present a new theoretical foundation for the phyloAge* approach and a flexible new model for estimating blood diversity decay which can be customized for application to bulk-seq data. We first tested it on 157 individuals with Acute Myeloid Leukemia from The Cancer Genome Atlas (TCGA-AML), which we used to test for accelerated physiological aging in individuals with AML, as reported in Craig et al. (2024). We then used a second cohort to test the hypothesis that mutations in some cancer driver genes can confer longevity (Wang et al. 2024). This was proposed because CH-promoting variants were found in a majority of long-lived individuals (90–110 years old) but were rare in younger individuals (65–80 years old) (Wang et al. 2024). Paradoxically, this could tie excesses of these variants to a lower risk of blood cancer in long-lived individuals. This hypothesis led us to predict that the HSC phyloAge* estimates of long-lived individuals would be lower than their chronological ages, because age directly correlates with blood cancer risk. This hypothesis can now be tested using the VFS phyloAge* model, as developed and applied for data collected by Wang et al. (2024), who reported somatic variants with > 1% frequency in a few CH-promoting loci. In the following, we present results showing acceleration and deceleration of HSC aging in patients and long-lived individuals, respectively.

## Results

### New Metrics for Estimating Diversity Decay

Bulk sequencing (bulk-seq) produces a sample of single nucleotide alterations (SNAs) and their variant allele frequencies (VAFs), constituting an individual’s somatic variant frequency spectrum (VFS). HSC proliferation, which characterizes CH, results in clusters of closely related secondary HSCs, each sharing one or more common variants due to shared ancestry and lack of recombination. As CH progresses, these variants accumulate, producing a detectable signal in bulk-seq data. For example, mutations on a phylogenetic lineage leading to CH, as indicated in red in Fig. 1b, will result in many variants with elevated and similar VAFs in the bulk-seq VFS (indicated by a red arrow in Fig. 1d). Assuming that every secondary HSC produced as a result of CH replaces an embryonic HSC, CH causes a reduction in phylogenetic diversity proportional to the product of the fraction (*f*) with which a cluster of variants occurs and the number of variants (*n*) in that cluster, i.e., *f* × *n*. Visually, *f* × *n* is the area of the white space in the HSC phylogeny in Fig. 1b. Thus, quantifying the shape of the VFS provides a practical means of assessing diversity decay from VAFs observed in the bulk-seq data.

Computational approaches are available to identify variant clusters in bulk-seq data (Roth et al. 2014; Chen et al. 2020; Khan and Mallory 2023). We used the standard analysis pipeline implemented in the software package PyClone (Roth et al. 2014) to infer distinct subclonal populations in bulk sequencing data by grouping SNAs into clusters based on the fraction of reads supporting the variant allele call (see Materials and Methods). PyClone estimates distinct subclonal clusters in the bulk sequencing dataset, which we used to calculate a new biodiversity decay metric (*γ*). Thus, *γ* captures the sum of the reduction in phylogenetic diversity caused by all the CH events, corresponding to the number of clusters produced by PyClone:

1 $$\gamma = \, \sum_{i} f_{i} \times n_{i}$$

where *f*_i_ is the frequency of cluster *i* identified by PyClone, and *n*_i_ is the number of variants in that cluster. The sum in Eq.1 is taken over all variant clusters, except a very large cluster of rare variants that all correspond to variants that arose on the tips of the HSC phylogeny (see Materials and Methods).

**Fig. 3:**
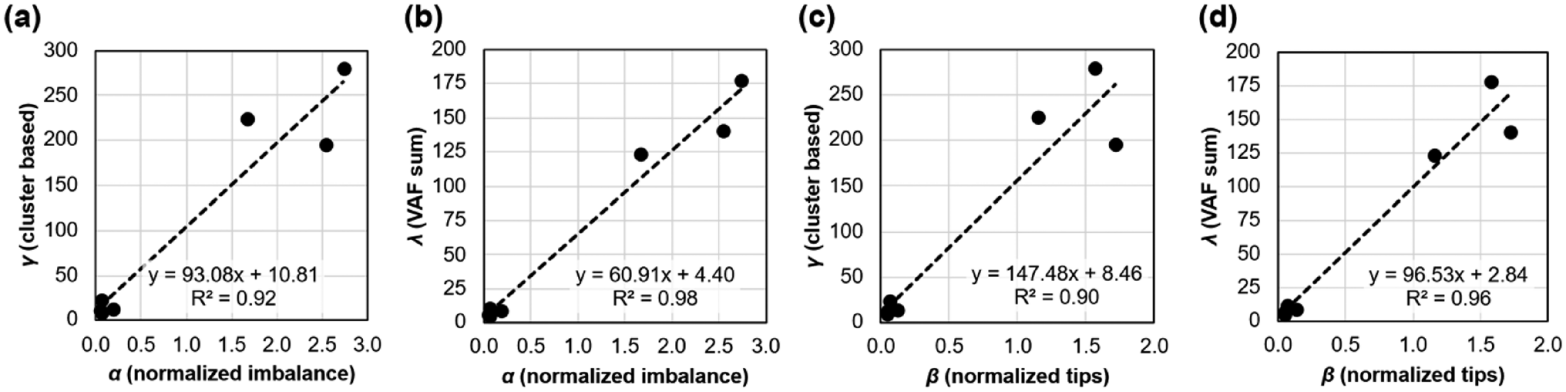
Relationship between phylogeny-based and VFS-based metrics of phylogeny decay in healthy individuals**: a** *α* and *γ*, **b** *α* and *λ, ***c** *β* and *γ*, and **d** *β* and *λ*. Dashed lines show the fit of a linear regression in each panel

To validate the PyClone-based *γ* metric, we examined its relationship with *α* and* β*, the phylogeny shape metrics developed by Craig et al. (2024). Here, *α* captures per-phylogeny normalized Colless’ (1982) imbalance, while *β* captures a normalized metric of overall HSC count. If the two are highly correlated with *γ*, we may conclude that *γ* is sensitive to the same pattern of diversity decay, but does not need the HSC phylogeny to recover this signal. For a direct comparison, we derived a somatic VFS for each individual using the colony-seq dataset (Mitchell et al. 2022) to estimate *γ*, since this dataset is required to estimate *α* (see Materials and Methods). We ran PyClone on colony-seq somatic VFS for healthy individuals and estimated *γ* using Eq.1. There was a high correlation between *α* and *γ* (Fig. 3a; *R*^2^ = 0.92) and between *β* and *γ* (Fig. 3c; *R*^2^ = 0.90), establishing that *γ* captures the same signal as phylogeny-based metrics. A high correlation was also observed when comparing *γ* estimated from the somatic VFS for MPN patients with *α* estimated from the collection of HSC genomes generated by colony-seq (*R*^2^ = 0.68) (Williams et al. 2022).

However, inferring variant clusters with PyClone was computationally intensive, taking days to run for some colony-seq datasets due to the large number of variants and the fact that many variants can have very similar VAFs (see Materials and Methods). To avoid this computational bottleneck, we developed a more streamlined measure of diversity decay (*λ*) in which:

2 $$f_{i} \times n_{i} \simeq \sum_{j} f_{i,j}$$

Then, *λ* can be calculated as the sum of VAFs over all the variants in all the clusters:

3 $$\lambda = \, \sum_{i} f_{i} \times n_{i} = \, \sum_{i} \sum_{j} f_{i,j}$$

Since every variant maps to only one cluster, Eq. 3 can be simplified as follows:

4 $$\lambda {\text{ }} = {\text{ }}\sum k {\text{ }}\mu _{k} {\text{ }}$$

Here, *μ*_k_ is the VAF of variant k, and the sum is taken over all the VAFs.

The application of Eq. 4 requires that all variants mapping to the tips of the HSC phylogeny be excluded. These tip-specific variants are expected to occur in a single HSC lineage out of hundreds sampled, so that they will carry frequencies of 1% or lower in the VFS. Thus, to adequately filter them from the VFS, we imposed a 1% VAF threshold, effectively removing any SNAs that occur on a single phylogenetic tip out of hundreds.

To validate *λ*, we compared it to the phylogeny-based *α* and cluster-based *γ* metrics. As before, we calculated both *λ* and *γ* from the colony-seq dataset (Mitchell et al. 2022). Phylogeny-based (*α* and *β*) metrics are all tightly correlated with VFS-based *λ* (Fig. 3b and d). Thus, the phylogeny-informed insight that VAF sums contain an inherent signal of CH allows us to dramatically reduce the computational bottleneck in estimating phyloAge* from VFS obtained via bulk sequencing.

### Building a Phyloage* Model Using Somatic VFS

**Fig. 4:**
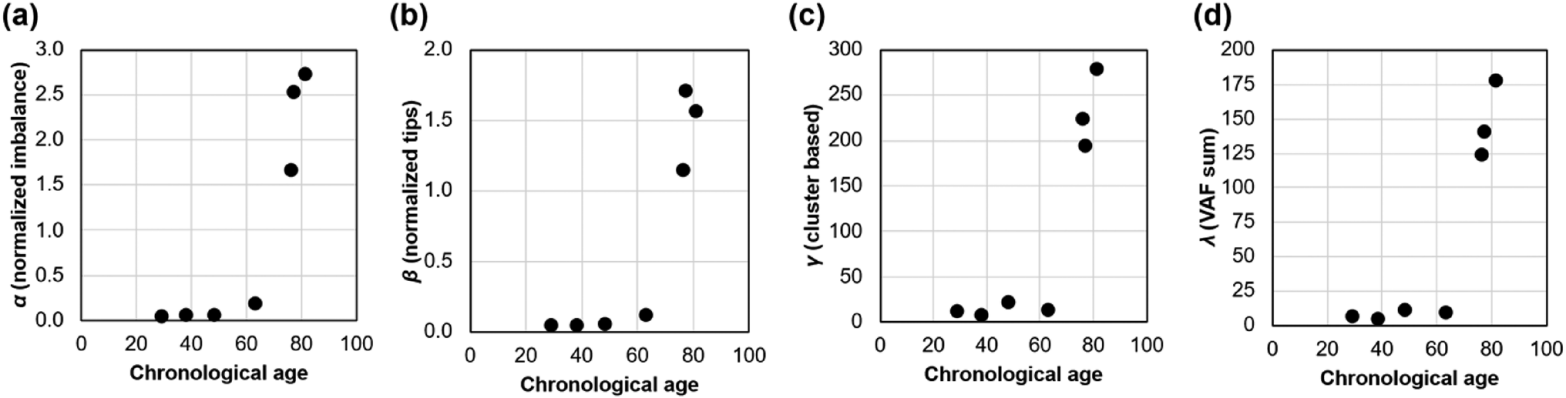
Relationship between age and different metrics of phylogeny decay: **a** *α*, **b** *β*, **c** *γ*, and **d** *λ*. All metrics increase exponentially with age

The VFS-based *λ* metric increases exponentially with age, just like phylogeny-based metrics (*α* and *β*), suggesting that it may be used in the same phyloAge modeling framework (Fig. 4). We developed a predictive model for estimating phyloAges from *λ* following the Craig et al. (2024) procedure:

5 $$phyloAge* \, = \, 1/b \times \, \left( {\log \, \left( \lambda \right) \, - a} \right)$$

where *a* and *b* are estimated using the relationship between *λ* and the chronological age of the healthy individuals. They were −1.65 and 0.084, respectively.

**Fig. 5:**
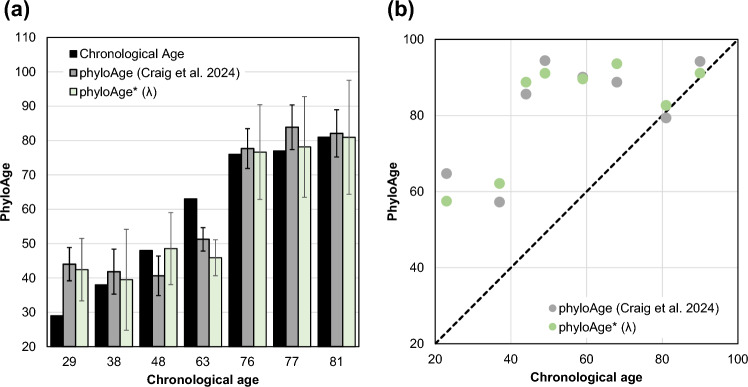
Validating estimates of phyloAge*. **a** Estimates of phyloAge* based on the VFS metric *λ* are similar to actual chronological ages, as well as to those based on the HSC phylogeny shape metrics *α* and *β.* **b** A comparison of phyloAge* estimates based on *λ* with those based on *α* and *β* for individuals with MPN

In a leave-one-out (LOO) analysis, on average, phyloAge* estimates differed by 4.9 years from the chronological ages of healthy individuals (Fig. 5a). The difference was biggest for younger individuals (average of 8.1 years for those under 65), who experienced low rates of HSC diversity decay and cancer incidence. The difference was relatively small among older individuals (an average error of 0.6 years for those aged 65 or older). These patterns are similar to those observed for the phyloAge model based on the HSC phylogeny-based approach (Fig. 5a).

To further validate the phyloAge* estimates, we computed root mean squared error (RMSE) and mean absolute error (MAE) for age-stratified bands of our reference dataset, covering individuals from 0–30 years old (RMSE = 8.3 and MAE = 4.9 years, respectively), 31–45 years old (1.5 and 1.5 years), 46–60 years old (0.5 and 0.5 years), 61–75 years old (17.1 and 17.1 years), and 76–100 years old (0.8 and 0.6 years). We also performed a simple calibration regression between chronological ages and phyloAge* estimates, yielding an intercept of 0.0 (−33.6–33.6), a slope of 1.0 (0.45–1.55), and an *R*^*2*^ of 0.81 (0.28–1.00). Taken together, this suggests that the phyloAge* estimates we report can be reliable despite the small sample size (Tables S1 and S2).

We also tested the performance of the phyloAge* model using somatic VFS derived from colony-seq HSC genomes of MPN patients. The application of Eq. 5 for these colony-seq VFS data produced excess HSC phyloAges similar to those produced using HSC phylogenies in Craig et al. (2024) (Fig. 5b). Thus, phyloAge* models based on VFS derived from accurate VFS variation data can be as effective as those in which single-cell HSC phylogenies are used.

### Building a Phyloage* Model Using Empirical Bulk-Seq Data

Our literature survey revealed that many cancer studies primarily apply bulk-seq techniques to detect tumor variants and estimate their VAFs in the coding regions in cancer patients. For example, many cohorts in The Cancer Genome Atlas (TCGA) include data on somatic variation in cancer patients. In these data, somatic variants in the blood bulk-seq data are often identified by reference to personal germline sequences. Estimating HSC physiological age using these data requires phyloAge* models built from somatic VFS data from healthy individuals, in which somatic variants are robustly identified, *e.g.*, using personal germline sequences. Unfortunately, we found no such data from TCGA or any other source for healthy individuals.

This prompted us to evaluate the feasibility of using somatic VFS derived from bulk sequencing of healthy (non-cancer) individuals, without germline sequencing of those same individuals. We analyzed somatic VFS for 147 healthy individuals aged 1–87 (see Materials and Methods). Variants were called using GATK4 MuTect2 (van der Auwera and O’Connor 2020). Somatic variants were detected by excluding variants found in the 1000 Genomes dataset and those present in the bulk-seq data of two or more of the 8,000 individuals from Ukraine (see Materials and Methods). Using the resulting somatic VFS, we evaluated the suitability of bulk-seq data by plotting the number of somatic variants identified against the chronological age, a pattern established in many previous studies (Lee-Six and Kent 2020; Mitchell et al. 2022; Craig et al. 2024). Unfortunately, the relationship was extremely noisy, and the trend was negative (see Fig. 9 in Materials and Methods). Indeed, it is well known that determining somatic mutations in bulk sequences alone is challenging without paired germline sequencing (Teer et al. 2017).

### Estimates of Phyloage* for the TCGA-AML Cohort

**Fig. 6:**
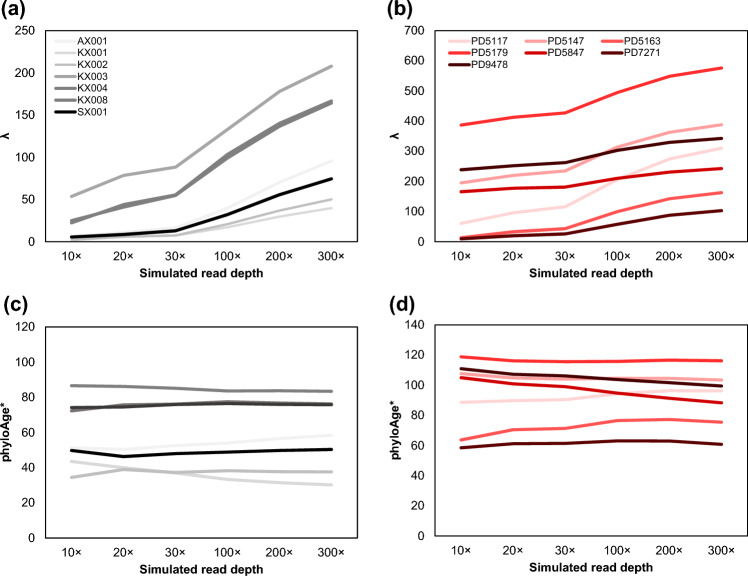
Response of* λ* and phyloAge* to read depth. For all seven healthy individuals (**a**) and seven with MPN (**b**), *λ* was calculated under six levels of simulated read depth (see Materials and Methods). Estimates of phyloAge* remained robust to read depth changes in both healthy individuals (**c**) and seven with MPN (**d**)

The lack of reliable somatic VFS datasets from healthy individuals led us to explore training a phyloAge model using somatic VFS derived from HSC genomes sequenced by colony-seq instead. However, the reported somatic variants for the TCGA-AML cohort are limited to coding sequences, unlike the genome-scale data available from colony-seq, which required building a phyloAge* model using colony-seq VFS restricted to the exome variants. In addition, TCGA-AML bulk-seq datasets are sequenced at an average of 30 × coverage (Ley et al. 2013), which is many-fold lower than the colony-seq data. So, the phyloAge* model needs to account for this coverage difference, as a direct relationship between *λ* and read depth was observed (Fig. 6a-b; see Materials and Methods). This is because at higher read depths, more low-frequency variants can be reliably detected, passing the 1% VAF cutoff and contributing to *λ.* Notably, the estimates of phyloAge* remained similar when the phyloAge* model accounted for the read depth (Fig. 6c-d).

**Fig. 7:**
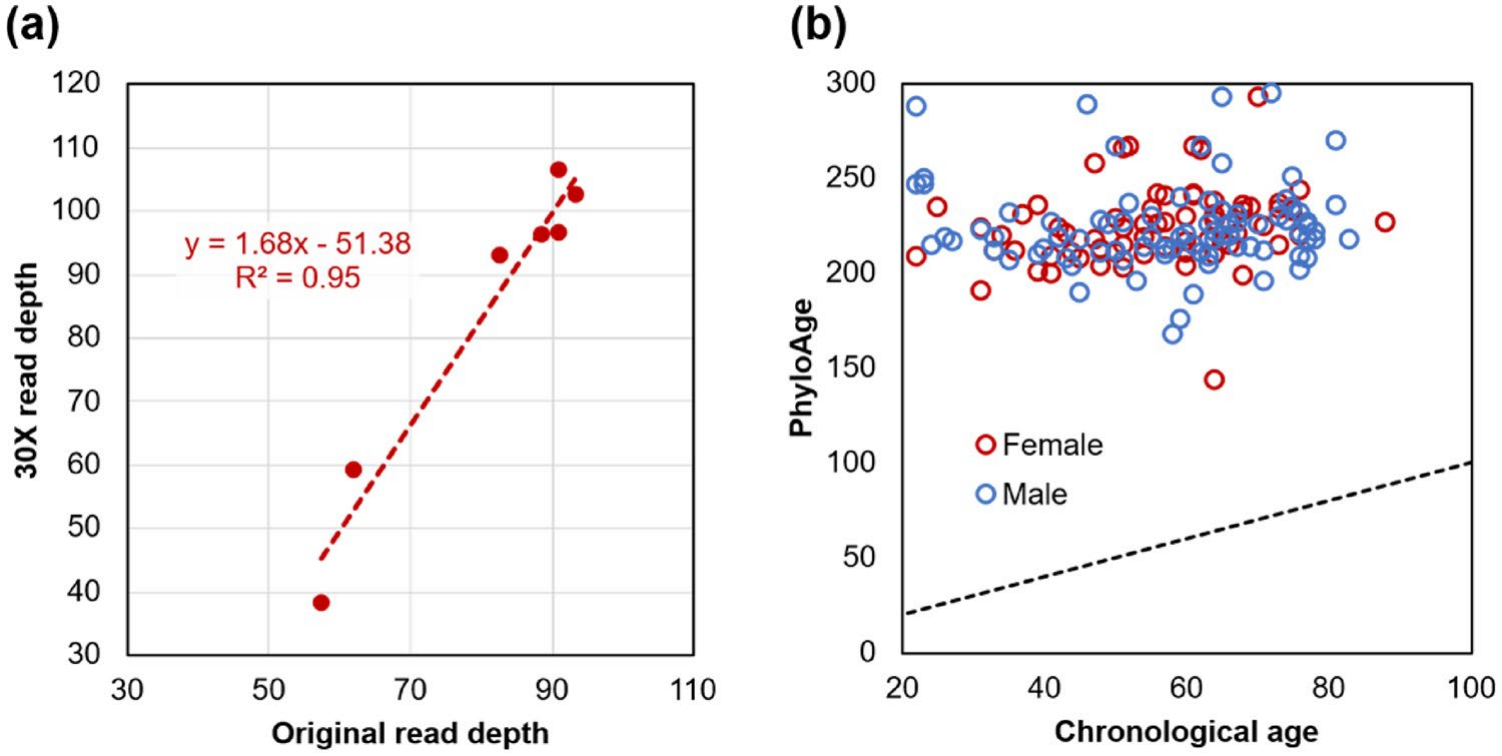
Estimates of phyloAge* for individuals with blood cancer. **a** Relationship of PhyloAge* estimates for individuals with MPN inferred at a simulated 30 × read depth with those estimated from the original data for all SNAs at coding sites. The correlation is *R*^*2*^ = 0.95 (*P* < 0.001). **b** The phyloAge* estimates for 157 individuals from the TCGA-AML cohort are plotted against their chronological ages. Circle colors denote the patient’s sex: male (blue) and female (red). The dashed line marks a 1:1 relationship

Given the robustness of phyloAge* to read depth in simulation, we performed an empirical validation by building a customized exome-phyloAge* model (30 × coverage) and tested it using exomic VFS (30 × coverage) derived from HSC genome datasets of MPN patients. The phyloAge* estimates were very similar to those obtained using the original, high-read-depth data (*R*^*2*^ = 0.95, *P* < 0.001; Fig. 7a). We then used this exome-phyloAge* model to estimate HSC phyloAge* for 157 TCGA-AML individuals aged 18–83 at the time of the bulk-seq profiling (Fig. 7b). PhyloAges were consistently elevated (143–294, mean = 223) compared to chronological ages of patients (18–83, mean = 57), with an average residual age of 168 years. This difference is statistically significant (*P* < 0.001). No significant difference in trend was observed between male and female patients (t-test, *P* > 0.90). Thus, we conclude that the exome-phyloAge* model trained on somatic VFS is sensitive to increased phylogeny decay in individuals with blood cancers, while being robust to changes in read depth.

### Estimates of Phyloage* for Long-Lived Individuals

Next, we developed a customized phyloAge* model for contrasting the HSC physiological ages of a cohort of long-lived individuals (90–110 years old) with those of younger individuals (65–89 years old) (Wang et al. 2024). The VFS data consisted of variant frequencies across 46 CH-associated cancer driver genes. As above, we restricted the colony-seq VFS to these 46 markers to normalize coverage area, developed a Wang-phyloAge* model, and then applied it to bulk-seq somatic VFS of 113 individuals (see Materials and Methods). To validate the Wang-phyloAge* model, we compared its results to those from the phyloAge* model based on the entire somatic VFS. It showed a moderate relationship (*R*^2^ = 0.69) due to larger overestimates for younger individuals.

**Fig. 8:**
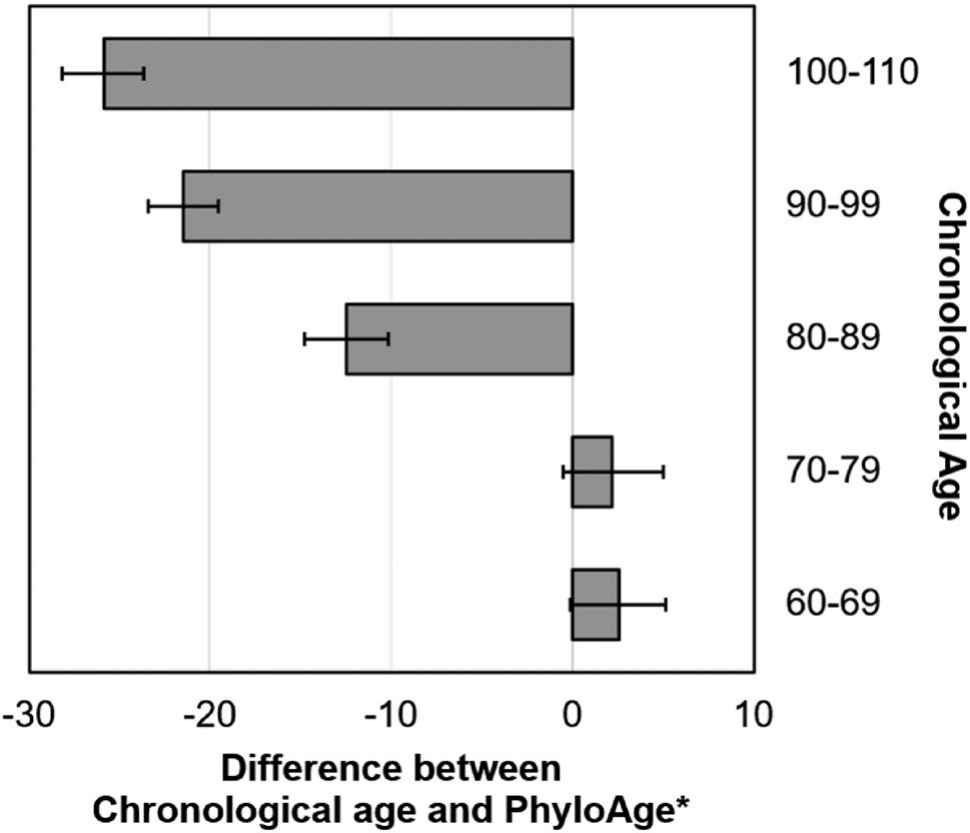
Estimates of phyloAge* for long-lived individuals. The difference between chronological age and phyloAge* for different age cohorts in the Wang et al. (2024) dataset. Whiskers show 95% confidence intervals around the mean. Negative values indicate lower phyloAges than the chronological ages. Differences are statistically significant (*P* < 0.05) for groups of individuals above 80 years of age

The Wang-phyloAge* model produced similar estimates for HSC phyloAges and chronological ages of individuals aged 60–79 of the cohort (Fig. 8). This could be taken to suggest that the Wang-phyloAge* model works well for individuals within a typical healthy lifespan. By contrast, estimated phyloAges* were lower than chronological ages in long-lived individuals (*P* < 0.001), with this difference increasing with age (Fig. 8). These patterns are consistent with our hypothesis of reduced blood risk with age in long-lived individuals.

This result allows us to test the counterintuitive hypothesis that mutations in certain known cancer driver genes can confer longevity. This was suggested by Wang et al. (2024) who reported that variants in genes like *TET2* are found much more often in long-lived individuals as compared to individuals ages 60–79 years old, despite their known association with cancer (Abelson et al. 2018; Desai et al. 2018). We predicted that long-lived individuals would have lower phyloAge* estimates than their chronological ages if longevity is significantly impacted by lower cancer risk. Indeed, healthy individuals 80–110 years old had phyloAge* estimates significantly lower than their chronological ages (Fig. 8).

The phenomenon of stem cell exhaustion could offer a possible explanation for this surprising pattern (Geiger et al. 2013; He and Wang 2021). As individuals age, some of their HSCs tend to lose potency, reducing the total number of viable HSC lineages in the blood and the capacity of the blood to generate newly differentiated blood cells (Ruzankina and Brown 2007; Jacob and Osato 2009). This shortfall may be overcome by increased HSC production driven by CH factors, thereby promoting cell division. Thus, mutations that drive the expansion of secondary HSCs may provide benefits by increasing HSC count in such circumstances, in contrast to their detrimental impact when they occur earlier in life and displace existing, healthy HSCs (Wang et al. 2024). Any causal link between longevity and variants in cancer-associated genes, such as *TET2*, late in life remains to be proven and represents a compelling open question.

### Estimates of Phyloage* for Multiple Samples from Individuals

Treatment for blood cancers has been shown to directly impact HSC clonal diversity (Uryu et al. 2024), so we would expect to see a change in phyloAge* estimates pre- and post-treatment. While we found no high-quality datasets suitable for estimating and comparing the phyloAge* of multiple samples from the same individual over a long time, Williams et al. (2022) reported four cases in which an individual with MPN was resampled after interferon-alpha (IFN) treatment. Of these, three (PD6646, PD6629, and PD4781) were resampled within a few years of their initial sampling, and showed only marginal changes in phyloAge* estimates (increases of 1.0, 3.9, and 0.4 years, respectively), while the difference between phyloAge* and chronological age remained high (15 years or more).

However, one individual (PD5182) was sampled three times (32, 46, and 53 years of age). The first sample, collected before IFN treatment at age 32, showed an excess in phyloAge* of more than 38 years. The patient underwent surgery, and remarkably, in their next sample, this excess phyloAge* was reduced to just 19 years. That is, more than a decade after IFN treatment, their phyloAge* had not grown, but in fact declined. This is likely because IFN targets HSCs undergoing CH, thereby reducing the preponderance of CHs and improving phyloAge*. A subsequent HSC sample, obtained 6 years later at age 53, yielded a phyloAge* of 78, an excess of only 25 years. This represents a promising but anecdotal result which we hope will encourage the collection of larger high-quality datasets in the future.

### Comparison of *λ* with Other Metrics of Population Diversity

The phyloAge* approach introduced here has conceptual links to several prior metrics of diversity in population genetics. First, the *λ* metric we develop is most closely related to our previous *α* and *β*, as they share a strong conceptual framework in quantifying observable change in phylogeny shape. Second, Mitchell et al. (2022) derived a metric of Shannon diversity (*ShD*) based on the number of phylogenetic branching events among HSCs after the embryonic phase, taking a threshold number of novel variants as a proxy for the end of embryonic development. Application of these three metrics requires an HSC phylogeny, unlike *λ*.

*λ* also shares a conceptual relationship with Hill numbers, where the first Hill number (q = 0) captures species or allelic diversity, the second (q = 1) captures Shannon entropy, and the third (q = 2) captures Simpson concentration. All three of these metrics quantify aspects of a population’s genetic diversity, whereas *λ* quantifies the loss of this diversity with the incidence of CH. However, calculating Hill numbers requires clonal structure inference, such as with PyClone. Traditional metrics developed to quantify diversity from multilocus allele frequencies, such as within-population average heterozygosity, *π* (Nei 1973), may be calculated from the VFS directly. Other metrics, such as Tajima’s *D* (Tajima 1989) and Fay and Wu’s *H* (Fay and Wu 2000), are calculated using sequence alignments, rather than VFS. In addition, the progression of AML has been quantified by treating the largest VAF as a proxy for excess CH (Toth et al. 2019).

We estimated all these metrics for our samples of healthy individuals and those with MPN, using HSC alignments (*D* and *H* metrics) and phylogenies (*ShD*, *α*, and *β*), as needed for making comparisons. We performed correlation and linear regression analyses (Table 1), and found a strong correlation between phylogeny-based metrics (*α* and *β*) and *λ*, which is desirable as *λ* was designed to capture phylogenetic signals without a phylogeny. We also detected a modest correlation with *ShD* that could not be computed from bulk data. The correlation with *D* and *H* metrics was lower (0.78 and 0.74 in healthy individuals), as was the correlation with the Toth et al. (2019) metric (0.69 in healthy individuals). The latter metric considers variants involved in the most frequent CH, ignoring many others (see Fig. 1b), which may be the reason for its inability to predict myeloblast prevalence reported previously (Toth et al. 2019). Finally, our attempts to develop a predictive model using the framework in Eq. 5 did not succeed, as these metrics do not show the relationship trends with chronological age that were evident using *λ*. In any case, most of them could not be calculated from the VFS alone, as they required clonal population structure inference, sequence alignments or phylogenies.

**Table 1:** Correlation coefficients (*R*^*2*^) reported from regressions between the new *λ* metric and previous metrics

	**Correlation with *λ***	
**Metric**	**Healthy**	**MPN**
***α* (normalized imbalance)**	0.98	0.74
***β* (normalized tips)**	0.96	0.75
***ShD* (** Mitchell et al. 2022**)**	0.95	0.67
**Hill q = 0**	0.34	0.00
**Hill q = 1**	0.29	0.01
**Hill q = 2**	0.29	0.04
***γ* (PyClone)**	0.98	0.99
***π* (** Nei 1973**)**	0.82	0.70
**Largest VAF (** Toth et al. 2019**)**	0.69	0.32
**Tajima’s *D***	0.78	0.68
**Fay & Wu’s *H***	0.74	0.44

## Conclusions

Clonal dynamics among HSC lineages in an individual’s blood are increasingly being viewed through an evolutionary lens to better understand blood health during aging and pathology (Robertson et al. 2022; van Zeventer et al. 2023; Fabre and Vassiliou 2024). We have shown that novel approaches for estimating HSC diversity decay from blood bulk-seq data are useful for building models to assess changes in physiological HSC age relative to chronological age. Previously, phyloAge performed comparably to established physiological aging approaches, such as GrimAge2 (Lu et al. 2022) and DeepMAge (Galkin et al. 2021), which have been used to detect clonal expansion (Kreger et al. 2024). Here, we demonstrate that VFS-derived phyloAge* preserves that behavior in principle, producing comparable results for healthy individuals (where phyloAge is expected to match chronological age) to those reported with methylation clocks that rely on thousands of markers. Indeed, in preliminary tests, estimates of phyloAge* show comparable accuracy and discrimination between healthy individuals and those with blood cancer (see Fig. S1).

Furthermore, we can quantify HSC genomic diversity decay without needing HSC phylogenies derived from expensive high-resolution or high-coverage datasets (Fig. S2). In fact, the need for an HSC phylogeny originally precluded phyloAge estimation for the TCGA-AML and the cohort of long-lived individuals. We overcame this by recognizing the simple phylogenetic principle that any variants acquired by a given lineage will be inherited by its direct descendants, and therefore elevated VAFs will capture the signal of phylogenetic splitting. Therefore, the sum of elevated VAFs is an effective metric of excess HSC lineage division due to CH. Importantly, as with phylogeny shape-based metrics, this approach is agnostic to HSC population size or mutation rate, as it instead quantifies their contribution to an individual’s blood diversity on the basis of shared ancestry.

The advancements to the phyloAge method we introduce here open up many novel applications needed by the broader community of researchers and clinicians for assessing age via the analysis of blood. The new computational approach presented here requires only data from bulk blood sequencing and is largely agnostic to panel selection. This flexibility makes phylAge* a promising tool as we expect many more investigators to generate bulk sequencing data retrospectively and clinicians to produce new datasets, including large, age-stratified cohorts and even longitudinal bulk sequencing profiles for individuals.

We provide a tool for developing tailor-made phyloAge* models based on the data from Mitchell et al. (2022) on GitHub (https://github.com/kumarlabgit/phyloAge). This tool allows users to carry out all analyses in this study, starting from colony-seq data, subsetting by chromosome, gene, CpG site, or known driver, and scaling the read depth to suit their target comparison data, then training a tailor-made phyloAge* model and testing it against data of their choice.

## Materials and Methods

### Data Acquisition

#### Colony-Seq Cohorts

 HSC sequences for healthy people (neonates to 81 years old) and individuals with MPN (20 to 83 years old) were publicly available from Mitchell et al. (2022) and Williams et al. (2022). Infants were excluded from all analyses because they were still experiencing rapid HSC diversification. HSC sequencing was paired with a sampling of another tissue type, either peripheral blood cells, buccal epithelium, or T cells from the same individual, facilitating accurate deconvolution of somatic from germline variants.

#### TCGA-AML Cohort

We downloaded bulk sequencing samples for 157 members of the Acute Myeloid Leukemia cohort of The Cancer Genome Atlas (TCGA-AML) from the TCGA Research Network (https://www.cancer.gov/tcga). We considered only SNAs called by MuTect2. All variant and reference read counts were extracted from the VCF reports available from the TCGA resource.

#### Long-Lived Cohort

The data from a long-lived cohort was obtained from the supplementary information of Wang et al. (2024). They reported personal somatic variants with VAFs ≥ 1% for 237 blood samples, each representing a detected driver mutation in one of 133 unique individuals aged 65 to 110 years. Of these 133, only 113 contained SNAs or other point mutations in their small panel of genes. Using the annotations provided by Wang et al. (2024), we selected variants stemming from point mutations because the phyloAge* models are built using those variants. We also retained only the 46 genes used by Wang et al. (2024), not their additional six aging-associate markers. Although VAFs from Wang et al. (2024) were not explicitly normalized for CNAs, we can rule this out as a confounding factor, since excess CNAs would have the effect of increasing estimates of *λ*, yet the values we find are unexpectedly low.

#### The Bulk-Seq Cohort

We analyzed novel bulk-seq data of 147 individuals who reported a healthy BMI, no history of smoking, little to no alcohol consumption, and overall self-reported excellent or good health. For people over 60, we included people with self-reported health as "average" to boost their numbers, as people over 60 tend not to answer "excellent" to this question. These 147 individuals were selected from a collection of more than 8,000 individuals from a cross-sectional study of T1D patients and controls collected during 2022–2024 in Ukraine (genes.uzhnu.edu.ua). According to the approved IRB protocol, the data from this project may be published and shared for research use. This was explained to each participant, and written informed consent was obtained and kept at a biobank at Uzhhorod National University in Ukraine that manages this collection (genes.uzhnu.edu.ua) established previously by the Joint Operational Programme Romania-Ukraine 2014–2020 under “Partnership for Genomic Research in Ukraine and Romania”. Exome sequencing and genome-wide genotyping were conducted at Regeneron Genetics Center (RGC), funded by The Leona M. and Harry B. Helmsley Charitable Trust “A comprehensive study of T1D exomes” (Phase 1 & 2) where post-quality control sequencing was completed on all samples using Twist whole exome capture and “globally-representative” genotyping SNP arrays. Only raw sequencing data, with no other identifiers or phenotypes, were provided for this study.

**Fig. 9:**
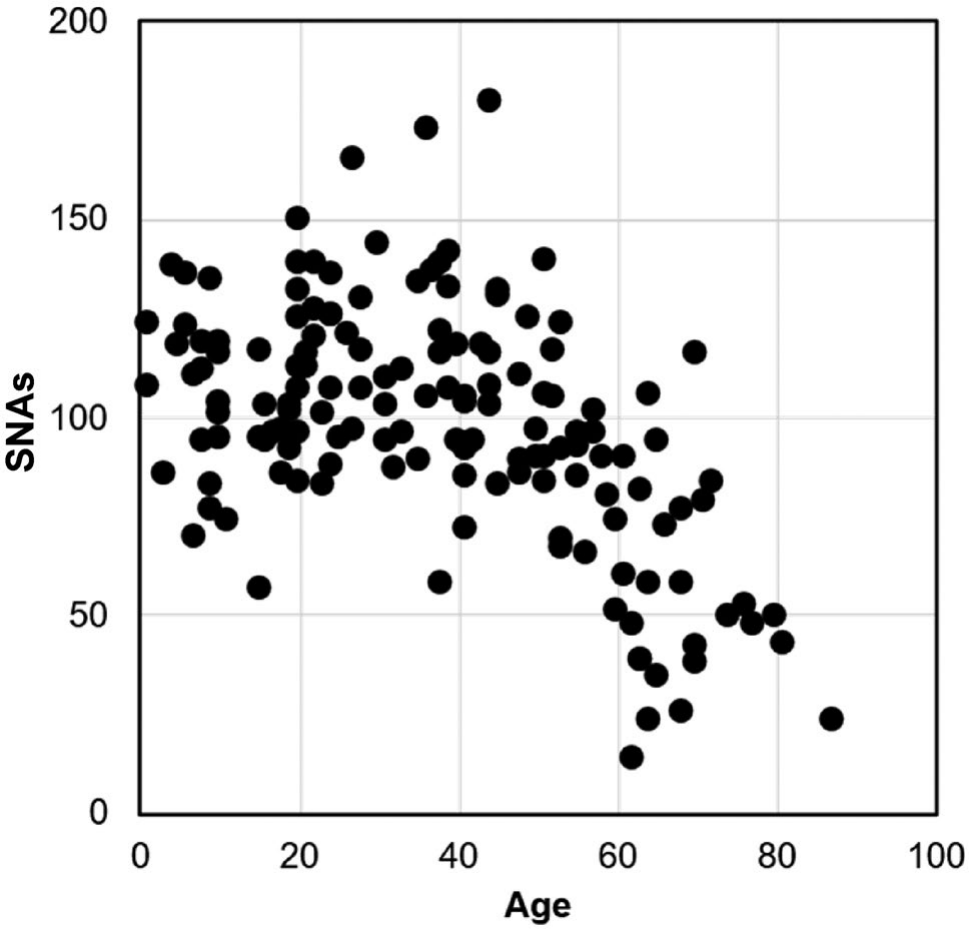
Relationship between age and the count of SNAs observed in the empirical bulk-seq datasets. The relationship is negative and significant (*P* < 0.00)

For all these individuals, somatic variant sites were called using GATK4 MuTect2 with PoN and filtering out all by the PASS tag. Given the ethnic homogeneity of this population, we further filtered potential germline variants by removing those detected in two additional somatic samples within the cohort. Finally, we excluded variants occurring at a site with a germline mutant occurring in any of the original 8,000 individuals, regardless of health status. This resulted in detecting an average of 39.8 variants per individual at an average read depth of 65.3 ×. Variants with frequencies less than 1% were discarded, as these reflect tip-lineage mutations and are not indicative of HSC genomic diversity decay (as noted earlier). There is a negative (albeit noisy) relationship between the number of variants and age (Fig. 9), which is likely an artifact because of the under-detection of variants with VAFs between 1–2%, which are near the detection limits of the average read depth of 65.3 ×, and occur with increasingly larger numbers with age (e.g., Fig. 2b vs. Figure 2c).

#### Deriving VFS from HSC datasets

Notably, the Mitchell et al. (2022) dataset we used as a reference for model training used a colony-sequencing approach, yielding a nearly complete collection of SNAs in every HSC cell sequenced. They first isolated 3,579 HSCs (224–453 per person) from blood via flow cytometry. Each HSC was individually cultured to produce a colony of 200–3,000 cells. Each cell in the colony was sequenced on the Illumina NovaSeq, generating 150 bp paired-end reads at an average read depth of 14 × per site. Pooling of 200–3000 cell sequences at 14 × average coverage per colony resulted in 2,800 × to 42,000 × coverage per HSC cell. This allowed for orders of magnitude more accuracy than typical single-cell sequencing at every site in every HSC genome. Consequently, only 0.5%–3.4% of sites had missing data across all HSC genomes.

From the somatic VFS data from colony-seq HSC genome collections, we estimated variant allele frequency (VAF) at each variant position as VAF = ½[mutant cell count/(non-mutant cell count + mutant cell count)]. The mutant read count at a given variant position was generated by scaling the observed total read count down to 30 reads (from > 300 ×) to simulate 30 × sampling. To scale the non-mutant read count, we simply subtracted the scaled mutant count from an assumed total count of 30. So, for a site with 300 total reads, we would divide the observed mutant count by 10 (300/30 = 10), then subtract this value from 30 to generate the non-mutant count.

### Clustering Variants Using PyClone

We ran the latest PyClone build (https://github.com/Roth-Lab/pyclone) in a Python 2.7 environment using the provided full-analysis-pipeline command. From the output, we extracted the “size” (number of variants) and “mean” (frequency) parameters from the resulting “cluster.tsv” table for each individual. PyClone analysis was performed under the assumption that variants were unaffected by copy-number alterations. So, we set normal and major copy numbers to 2 and minor copy numbers to 0 for all variants. Our datasets containing hundreds of thousands of variants took, on average, several days to run on a desktop PC with a 3.5 GHz processor and 128 GB of memory. We imposed a conservative 1% VAF threshold to remove variants that will likely map to the tip lineages in the HSC phylogenies before conducting PyClone analyses because single-cell sequences of only 100–300 HSCs were reported. In the output, we found that PyClone recovered a large cluster containing a vast majority of variants that occurred at relatively low frequency (< 4%), which is clearly spurious clustering, as those variants are generally mapped to different tips of the HSC phylogeny. So, they were excluded from further analysis.

### Building phyloAge* Models

#### Building a Model for Somatic VFS Derived from HSC Genomes

Using the somatic VFS derived from HSC genome collections obtained by colony-seq, we estimated an exponential age model for both *γ* and *λ* using Eq. 5. All models were trained using a log model within a meta-regression framework (Viechtbauer 2010) with a maximum-likelihood approach to infer the values of the constants *a* and *b* from initial values: *a* = 1/1000 and *b* = 0.3. In each case, we used the model to predict the physiological ages of the test individuals, including those from the MPN sample provided in the original publication (Williams et al. 2022) and the TCGA-AML cohort. We assessed the fit of each model using Root Mean Square Error and mean absolute error (RMSE and MAE), both overall and stratified by age (Table S1). We also carried out a simple calibration regression during training, resulting in a slope of 1.0 and an intercept of 0.0, suggesting a strong fit, and a bootstrapped *R*^*2*^ value of 0.81 (0.28–1.0), as expected given the sample size (Table S2). For the TCGA-AML cohort, male and female phyloAges were compared by two-tailed t-tests.

#### Building a Model for the TCGA-AML Cohort

The TCGA-AML data are from coding-region sequencing and have an average 30 × sequencing read depth. This prompted us to build an exome-phyloAge* model by deriving a 30 × sequencing coverage profile from the HSC genomes dataset, restricted to variants in coding regions (the reference data covered the full genome). Since *λ* is additive, this prevents imbalances due to differences in coverage. To do so, we drew a read depth (*r*) for each variant from a Poisson distribution with the mean equal to the read depth (*r* = 30). The number of mutant reads for a variant was drawn from a binomial distribution with *r* trials, and the rate of variant sampling was set to the VAF of that variant. While we found that *λ* increases predictably with *r* when we repeated this simulation at six levels of *r* (Fig. 6a-b), we confirmed that estimates of phyloAge* remain constant as long as the same read depth is enforced for both training and test phases of the modeling process (Fig. 6c-d). This is due to the inherent normalizing effect of the model training process, which aligns known age with observed *λ* values prior to prediction. We developed the exome-phyloAge* model using Eq. 5 and optimizing parameters *a* and *b* for the somatic VFS data which accounts for differences in read depth and coverage between the TCGA-AML cohort and the Mitchell et al. (2022) dataset.

#### Building a Model for the Long-Lived Cohort

Wang et al. (2024) generated VFS comprising all point mutations in a panel of 46 genes, so we extracted all SNAs at those same loci from the Mitchell et al. (2022) dataset. They excluded SNAs with a VAF of less than 1%, as we did, suggesting compatibility between the two datasets.

### Comparison of λ with Other Metrics

For comparison with *λ, α* and *β* were obtained from Craig et al. (2024). The phylogeny-based implementation of the Shannon index was reproduced from Mitchell et al. (2022), but necessitated modification, as their threshold for maturity was defined by the count of accumulated SNAs, which is sensitive to sequencing and variant calling. Instead, we plotted the lineages through time (LTT) plots for each phylogeny, and identified the inflection point where exponential lineage diversification plateaued, typically between two to three years of age. The three Hill numbers (species count, Shannon diversity, and Simpson concentration) were all derived from clusters identified by PyClone, as was the *γ* metric. Nei’s (1973) *π* metric of within-population average heterozygosity, the approach proposed by Toth et al. (2019) were estimated based on VAFs estimated from read counts provided by Mitchell et al. (2022). To calculate the alignment-based metrics, Tajima’s *D* (Tajima 1989) and Fay & Wu’s *H* (Fay and Wu 2000), we reconstructed binary alignments from variant data provided in Mitchell et al. (2022). All comparisons with *λ* were done by simple linear regression, with the correlation coefficient (*R*^*2*^) reported) in Table 1.

## Supplementary Information

Below is the link to the electronic supplementary material.

Supplementary file1 (XLSX 20 KB)

Supplementary file2 (DOCX 1780 KB)

## Data Availability

HSC sequences for healthy people (2022) and individuals with MPN (Williams et al. 2022) are publicly available. The incidence of Leukemia by age is available from Cancer Research UK (2016–2018, ICD-10 C91-C95). These data were used as-is in our analyses. The datasets used by Wang et al. (2024) are available as supplementary information in that article. The results published here are in whole or in part based upon data generated by the TCGA Research Network: https://www.cancer.gov/tcga. The data from “A comprehensive study of T1D exomes” (Phase 1 & 2) can be accessed at https://genes.uzhnu.edu.ua/genes-dashboard/. A tool for developing tailor-made phyloAge* models based on the data from Mitchell et al. (2022) can be found at https://github.com/kumarlabgit/phyloAge.
