# Supplementary material for: An Evolutionary Metric for Estimating PhyloAges from Bulk Sequencing of Hematopoietic Stem Cells Reveals the Tempo of Blood Aging in Cancer and Longevity
Source: J Mol Evol. 2025 Dec 26;94(1):177–89. doi: 10.1007/s00239-025-10296-y (PMC12920717; doi:10.1007/s00239-025-10296-y)
Supplement: Supplementary file 2 — Supplementary file2 (DOCX 1780 KB) [file 239_2025_10296_MOESM2_ESM.docx]

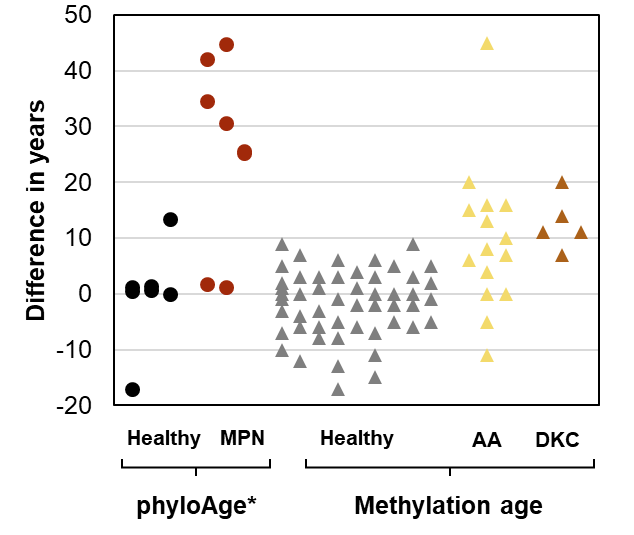


**Figure S1**: **Comparison with methylation-based physiological age.** Estimates of phyloAge* show comparable accuracy for healthy people (low difference between chronological age and predicted age in years) and comparable or better discernment between healthy individuals and those with blood cancer (measured as a high difference between chronological age and predicted age). Data points were retrieved from **Fig 4c** in Weidner et al. [(2014)](https://paperpile.com/c/TtWyt8/vMcO/?noauthor=1) (triangular markers) and redrawn with bulk-seq phyloAge* estimates from this study added (circular markers).


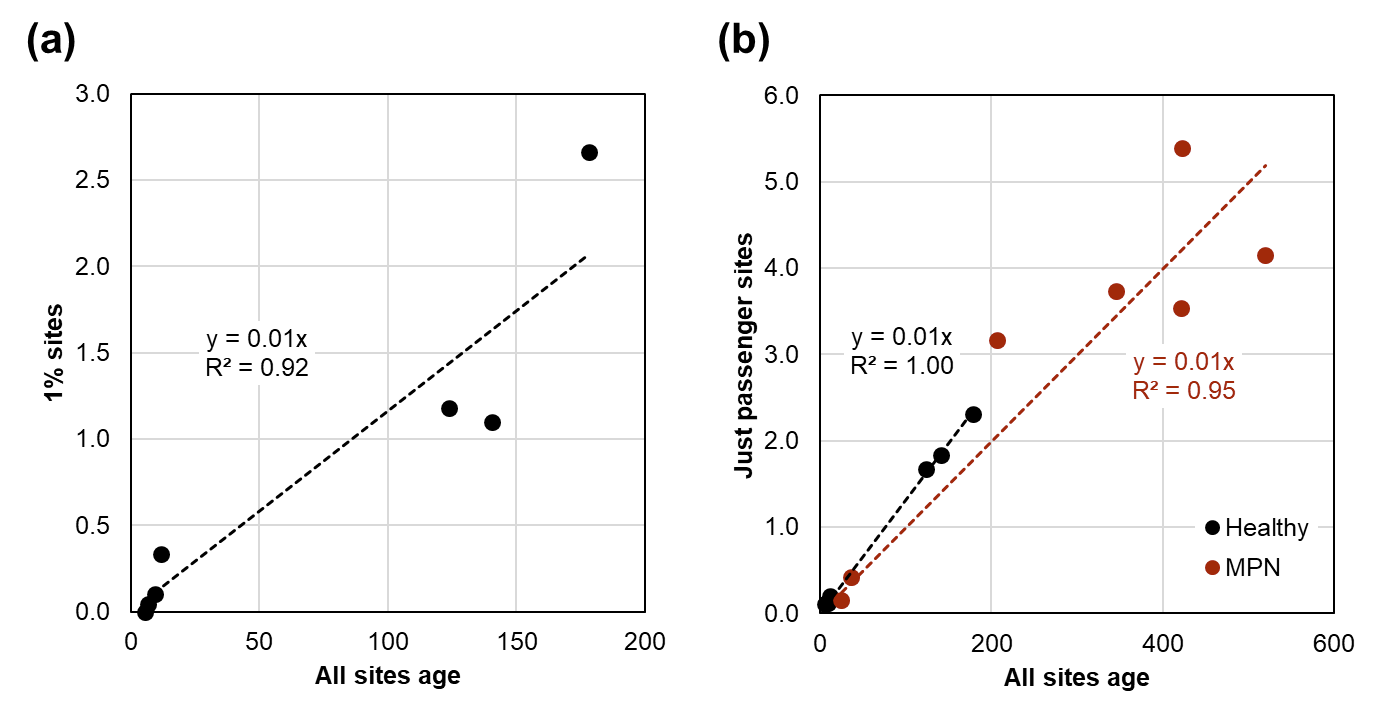


**Figure S2**: **Robustness of *λ* to coverage area and panel selection**. **(a)** The value of *λ* calculated from just 1% of all SNAs chosen at random is tightly correlated with that calculated from all SNAs for seven healthy individuals, demonstrating robustness to coverage area. **(b)** The value of *λ* calculated from only passenger sites (all SNAs occurring in CH-associated driver genes were dropped) is tightly correlated with that calculated from all SNAs for seven healthy individuals. This trend remains even when considering seven individuals with MPN, demonstrating robustness to panel selection even in cases of blood cancer. Driver genes here are defined as those with inferred CH-associated effects in Mitchell (2022) and Williams et al (2022) plus eight additional publications [(Papaemmanuil et al. 2013; Genovese et al. 2014; Link and Walter 2016; Bick et al. 2020; Feusier et al. 2021; Studd et al. 2021; Pich et al. 2022; Kishtagari et al. 2024)](https://paperpile.com/c/TtWyt8/qD5a+aioQ+sbY1+vrJW+Md39+bLbZ+0ZZT+04PD). All 151 drivers identified in at least one source are compiled in **supplemental data file S1.**

**Table S1:** Metrics of error in training phyloAge* models.


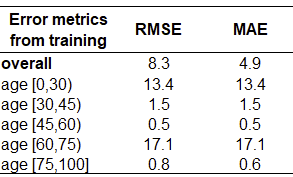


**Table S2:** Results of calibration regression performed in training phyloAge* models.


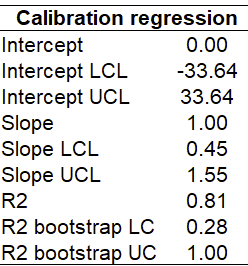


**Supplemental References**

[Bick AG, Weinstock JS, Nandakumar SK, et al (2020) Inherited causes of clonal haematopoiesis in 97,691 whole genomes. Nature 586:763–768](http://paperpile.com/b/TtWyt8/aioQ)

[Feusier JE, Madsen MJ, Avery BJ, et al (2021) Shared genomic segment analysis in a large high-risk chronic lymphocytic leukemia pedigree implicates in inherited risk. J Transl Genet Genom 5:189–199](http://paperpile.com/b/TtWyt8/sbY1)

[Genovese G, Kähler AK, Handsaker RE, et al (2014) Clonal Hematopoiesis and Blood-Cancer Risk Inferred from Blood DNA Sequence. N Engl J Med 371:2477–2487](http://paperpile.com/b/TtWyt8/qD5a)

[Kishtagari A, Khan MAW, Li Y, et al (2024) Driver mutation zygosity is a critical factor in predicting clonal hematopoiesis transformation risk. Blood Cancer J 14:6](http://paperpile.com/b/TtWyt8/vrJW)

[Link DC, Walter MJ (2016) “CHIP”ping away at clonal hematopoiesis. Leukemia 30:1633–1635](http://paperpile.com/b/TtWyt8/04PD)

[Papaemmanuil E, Gerstung M, Malcovati L, et al (2013) Clinical and biological implications of driver mutations in myelodysplastic syndromes. Blood 122:3616–27; quiz 3699](http://paperpile.com/b/TtWyt8/Md39)

[Pich O, Reyes-Salazar I, Gonzalez-Perez A, Lopez-Bigas N (2022) Discovering the drivers of clonal hematopoiesis. Nat Commun 13:4267](http://paperpile.com/b/TtWyt8/bLbZ)

[Studd JB, Cornish AJ, Hoang PH, et al (2021) Cancer drivers and clonal dynamics in acute lymphoblastic leukaemia subtypes. Blood Cancer J 11:177](http://paperpile.com/b/TtWyt8/0ZZT)

[Weidner CI, Lin Q, Koch CM, et al (2014) Aging of blood can be tracked by DNA methylation changes at just three CpG sites. Genome Biol 15:R24](http://paperpile.com/b/TtWyt8/vMcO)
